# Racial differences in *IGF1* methylation and birth weight

**DOI:** 10.1186/s13148-015-0080-6

**Published:** 2015-04-21

**Authors:** Jennifer K Straughen, Levent Sipahi, Monica Uddin, Dawn P Misra, Vinod K Misra

**Affiliations:** Department of Family Medicine and Public Health Sciences, The Wayne State University School of Medicine, 3939 Woodward Avenue, Detroit, MI 48201 USA; Center for Molecular Medicine and Genetics, The Wayne State University School of Medicine, 540 East Canfield, Detroit, MI 48201 USA; Department of Psychology, University of Illinois at Urbana-Champaign, 603 E. Daniel Street, Champaign, IL 61820 USA; Department of Pediatrics, Division of Genetic and Metabolic Disorders, The Wayne State University School of Medicine, 3901 Beaubien Blvd, Detroit, MI 48201 USA; Children’s Hospital of Michigan, Division of Genetic and Metabolic Disorders, The Wayne State University School of Medicine, 3950 Beaubien Blvd, Detroit, MI 48201 USA; Current address: Department of Public Health Sciences, Henry Ford Hospital, One Ford Place, Detroit, MI 48202 USA

**Keywords:** Birth weight, IGF1, Methylation, Epigenetics, Race, Disparities, Perinatal

## Abstract

**Background:**

The birth weight of Black neonates in the United States is consistently smaller than that of their White counterparts. Epigenetic differences between the races may be involved in such disparities. The goal of these analyses was to model the role of *IGF1* methylation in mediating the association between race and birth weight. Data was collected on a cohort of 87 live born infants. *IGF1* methylation was measured in DNA isolated from the mononuclear fraction of umbilical cord blood collected after delivery. Quantitative, loci-specific methylation was assessed using the Infinium HumanMethylation27 BeadArray (Illumina Inc., San Diego, CA). Locus specific methylation of the *IGF1* CpG site was validated on a subset of the original sample (*N* = 61) using pyrosequencing. Multiple linear regression was used to examine relationships between *IGF1* methylation, race, and birth weight. A formal mediation analysis was then used to estimate the relationship of *IGF1* methylation to race and birth weight.

**Results:**

Black race was associated with a 7.45% decrease in gestational age-adjusted birth weight (aBW) (*P* = 0.04) and Black infants had significantly higher *IGF1* methylation than non-Black infants (*P* < 0.05). A one standard deviation increase in *IGF1* methylation was associated with a 3.32% decrease in aBW (*P* = 0.02). Including *IGF1* methylation as a covariate, the effect of Black race on aBW was attenuated. A formal mediation analysis showed that the controlled direct effect of Black race on aBW was −6.26% (95% CI = −14.15, 1.06); the total effect of Black race on *IGF1* methylation was −8.12% (95% CI = −16.08, −0.55); and the natural indirect effect of Black race on aBW through *IGF1* methylation was −1.86% (95% CI = −5.22, 0.18)

**Conclusion:**

The results of the mediation analysis along with the multivariable regression analyses suggest that *IGF1* methylation may partially mediate the relationship between Black race and aBW. Such epigenetic differences may be involved in racial disparities observed in perinatal outcomes.

## Background

Racial differences in birth outcomes and their health consequences remain among the most persistent and vexing disparities in the United States [1]. For example, Black infants have consistently been shown to have smaller birth weights than their White counterparts [1]. Growing evidence suggests that such perinatal factors can impact health across the life course, including an increased risk of higher blood pressure, insulin resistance and diabetes, abnormal cholesterol profiles, pathologic patterns of fat deposition, and an elevated risk of coronary vascular disease later in life [2]. There is growing evidence that epigenetic changes may mediate the effects of the prenatal environments on short- and long-term health outcomes of the offspring [3].

DNA methylation is an important epigenetic mechanism that helps regulate gene expression and can be influenced by both the environment and the genome. DNA methylation has also been linked to some cancers, complex diseases, and transgenerational effects, and may serve as a potential link between the genome, environment, and disease [4-7]. On a population level, DNA methylation profiles differ across African and European ancestral groups [8]. For example, both at birth and in adulthood, African-Americans have lower genome-wide levels of methylation compared with individuals of European ancestry [9,10]. Such differences may contribute to health disparities observed between the two groups across the life course [11,12]. However, surprisingly few studies have explored racial differences in DNA methylation at birth [9].

Among epigenetically regulated genes, the insulin-like growth factors, including *IGF1* and *IGF2*, have particularly important roles in fetal and placental growth [13-17]. Loss of *Igf1* expression is associated with decreased fetal growth in mice [13]. Similarly, IUGR in the rat was associated with altered epigenetic characteristics of the *Igf1* gene, altered *Igf1* expression, and reduced IGF1 levels in liver and blood [16]. Partial deletion of *IGF1* in humans has been associated with impaired pre- and postnatal growth [15], while growth-restricted infants have been shown to have lower umbilical cord blood levels of IGF1 compared to their counterparts with normal growth [17].

The well-established effects of *IGF1* on birth weight along with persistently reported racial differences in birth weight and in genome-wide methylation studies have led us to examine the role of *IGF1* methylation in the pathway between race and birth weight. We hypothesized that *IGF1* methylation may partially explain racial differences in birth weight by mediating the association between race and birth weight. Indeed, our analyses demonstrated that *IGF1* methylation partially mediates the relationship between Black race and gestational age-adjusted birth weight (aBW). These results suggest that such epigenetic differences may be involved in racial disparities observed in perinatal outcomes.

## Results

Ninety mother-infant pairs were eligible for inclusion in this analysis. The analysis is restricted to 87 mother-infant pairs for whom race is known. Demographic and health characteristics of the 87 infants and their mothers are presented in Table 1. Approximately 24% of the mothers were Black and 76% were non-Black. Black and non-Black mothers were similar for several of the demographic and clinical characteristics considered. A majority of the women had adequate prenatal care and took prenatal vitamins. However, Black mothers were slightly younger than non-Black mothers (mean age 25.6 and 29.2 years, respectively; *P* = 0.02). While there were not any statistically significant differences in gestational age between the two groups, Black infants had a smaller mean gestational age-adjusted birth weight (*P* = 0.04).

**Table 1:** **Maternal and infant characteristics of the cohort by race**

	**Non-Black**		**Black**		
	***N***	**%**	***N***	**%**	***P*** **value**
Sample Size	66	75.9	21	24.1	
Adequate prenatal care					
No	13	19.7	6	28.6	0.38
Yes	53	80.3	15	71.4	
Parity					
Multiparous	43	65.2	16	76.2	0.43
Nulliparous	23	34.9	5	23.8	
Prenatal vitamin use					
No	10	15.2	6	28.6	0.20
Yes	56	84.9	15	71.4	
Smoker					
No	58	87.9	18	85.7	0.72
Yes	8	12.1	3	14.3	
Infant gender					
Female	28	42.4	11	52.4	0.46
Male	38	57.6	10	47.6	
	**Mean ± SD**		**Mean ± SD**		
Maternal age (years)	29.2 ± 6.6		25.6 ± 4.6		0.02
Gestational age (weeks)	38.0 ± 2.4		38.4 ± 1.7		0.49
aBW (grams)	3294.2 ± 455.0		3064.5 ± 483.3		0.04
*IGF1* methylation	0.26 ± 0.05		0.29 ± 0.06		0.03

*Abbr*: aBW = gestational age-adjusted birthweight; SD = standard deviation.

As shown in Table 1, Black infants had significantly higher *IGF1* methylation when compared to their non-Black counterparts, (*P* = 0.03). In Table 2, we analyzed *IGF1* methylation and birth weight by maternal and infant characteristics. *IGF1* methylation did not differ by adequacy of prenatal care, parity, maternal vitamin use, smoking status, or infant gender. In contrast, birth weight and gestational age-adjusted birth weight were associated with both parity and infant gender as expected.

**Table 2:** **The relationship of** ***IGF1*** **methylation and birth weight to select maternal and infant characteristics**

	***IGF1*** **methylation**	**BW (grams)**	**aBW (grams)**
	**Mean ± ** **SD**	**Mean ± ** **SD**	**Mean ± ** **SD**
Adequate prenatal care			
No	0.27 ± 0.06	3060.8 ± 773.3	3161.4 ± 500.5
Yes	0.26 ± 0.05	3274.6 ± 623.5	3260.3 ± 462.2
Parity			
multiparous	0.27 ± 0.05	3381.8 ± 531.6**	3331.7 ± 451.3**
nulliparous	0.26 ± 0.06	2903.8 ± 787.8	3042.8 ± 454.1
Prenatal vitamin use			
No	0.27 ± 0.05	3135.3 ± 568.5	3183.1 ± 343.9
Yes	0.26 ± 0.06	3248.8 ± 681.1	3251.2 ± 494.7
Smoker			
No	0.27 ± 0.06	3245.7 ± 662.4	3253.0 ± 437.9
Yes	0.26 ± 0.05	3105.0 ± 662.3	3139.7 ± 668.0
Infant gender			
Female	0.26 ± 0.07	2959.2 ± 721.0**	3090.2 ± 442.6*
Male	0.27 ± 0.04	3446.3 ± 518.4	3359.4 ± 460.4
Maternal age			
<30	0.27 ± 0.06	3123.8 ± 758.5	3210.7 ± 490.2
≥30	0.26 ± 0.05	3382.6 ± 445.4	3280.3 ± 440.9

*Abbr*: BW = birth weight; aBW = gestational age-adjusted birth weight; SD = standard deviation.

*P < 0.05.

**P < 0.01.

**Figure 1:**
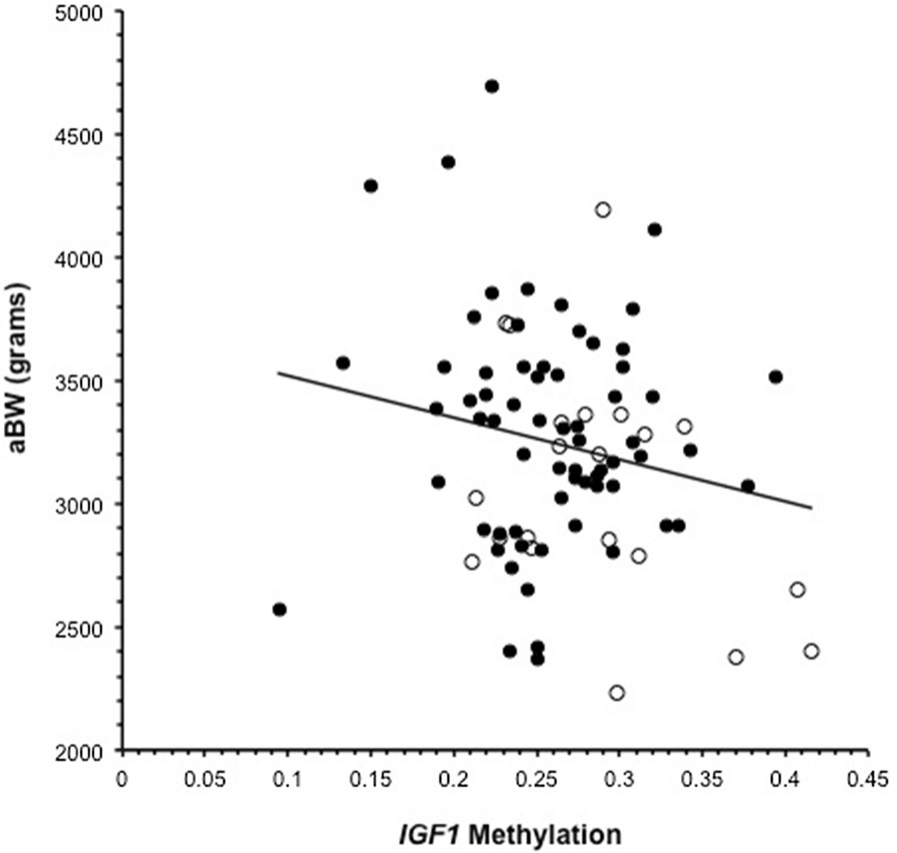
The relationship between gestational age-adjusted birth weight (aBW) and *IGF1* methylation. The open circles represent Blacks and the closed circles represent non-Blacks.

We modeled the relationships between race, *IGF1* methylation, and gestational age-adjusted birth weight using multivariable linear regression. Figure 1 shows the overall relationship of aBW to *IGF1* methylation depicting the individual values by race. Using regression models, *IGF1* methylation may be considered as a potential mediator of the relationship between race and aBW if several criteria are satisfied [18]: (a) there is a significant relationship between the independent variable (race) and the mediator (*IGF1* methylation); (b) there is a significant relationship between the mediator (*IGF1* methylation) and the outcome (aBW); (c) the independent variable (race) significantly affects the outcome (aBW); and (d) the effect of the previously significant relationship between the independent variable (race) and the outcome (aBW) is no longer significant when controlled for the mediator (*IGF1* methylation).

As shown in Table 3, Black race is associated with a 7.45% decrease in aBW (*P* = 0.04) after adjustment for maternal age, parity, and infant gender, and a 0.62 unit standard deviation increase in methylation of *IGF1* (*P* = 0.02) after adjusting for maternal age. A one standard deviation increase in *IGF1* methylation was associated with a 3.32% decrease in aBW (*P* = 0.02). The effect of Black race on aBW was attenuated and no longer significant (*β* = −5.68%, 95% CI = −12.84, 1.49) after including *IGF1* methylation as a covariate to the model. Thus, these results suggest that *IGF1* methylation may be a potential mediator of the relationship between race and aBW [18].

**Table 3:** **The relationships between race,** ***IGF1*** **methylation, and gestational age-adjusted birth weight** ^**1**^

	***IGF1*** **Methylation** ^**2**^			**% change in aBW** ^**3**^		
	***β***	**95% CI**	***P*** **value**	***β***	**95% CI**	***P*** **value**
Race						
Black race	0.62	0.11, 1.12	0.02	−7.45%	−14.44, −0.45	0.04
*IGF1* methylation						
Methylation z-score	-	-	-	−3.32%	−6.17, −0.47	0.02
Joint effects						
Black race	-	-	-	−5.68%	−12.84, 1.49	0.12
Methylation z-score	-	-	-	−2.70%	−5.63, 0.24	0.07

^1^Modeled using standard multivariable linear regression; ^2^ *IGF1* methylation z-score controlled for maternal age; ^3^gestational age-adjusted birth weight (aBW) controlled for maternal age, parity, and infant gender.

**Figure 2:**
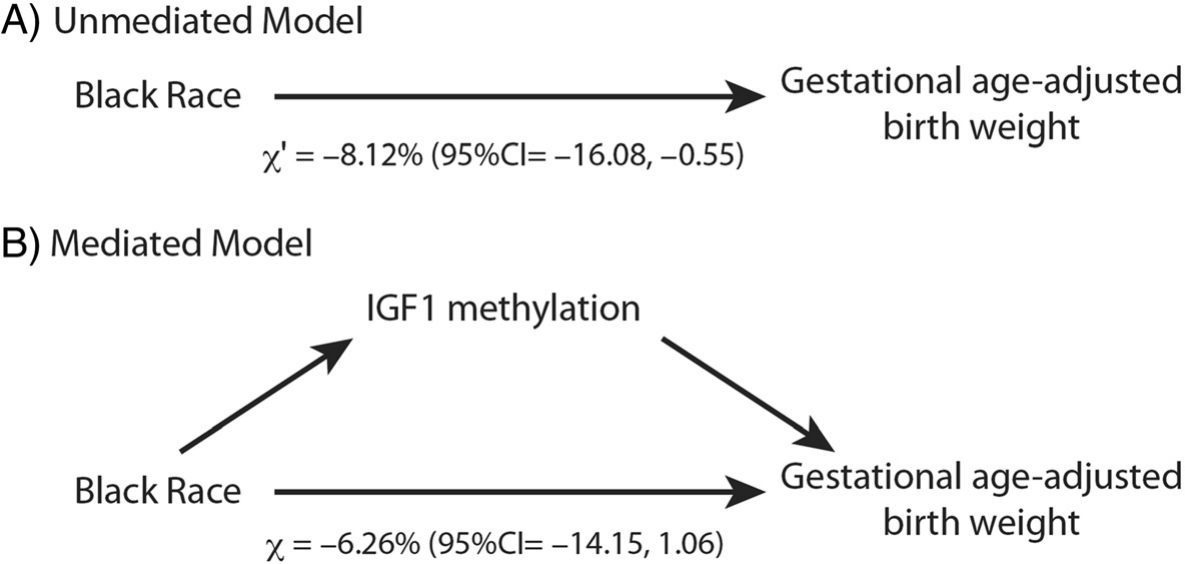
Models for mediation analysis for the relationship of Black race, *IGF1* methylation and gestational age-adjusted birth weight. **(A)** The unmediated model used to calculate the total effect. **(B)** The mediated model used to calculate the controlled direct effect. The calculated regression coefficients for the total effect, *χ*’, and the controlled direct effect, *χ*, are given.

As such, we used mediation analysis (see ‘Methods’) to formally test the hypothesis that *IGF1* methylation mediates the relationship between race and aBW as shown in Figure 2 [18]. These analyses were controlled for maternal age, parity, and infant gender. In this model, the *total effect* (Figure 2A) relating race and aBW was −8.12% (95% CI = −16.08, −0.55). The *controlled direct effect* relating race to aBW after adjusting for *IGF1* methylation status (Figure 2B) was −6.26% (95% CI = −14.15, 1.06). Using these results, the *natural indirect effect* was −1.86% (95% CI = −5.22, 0.18). The smaller direct effect (−6.26%) compared to the total effect (−8.12%) suggests that *IGF1* methylation partially mediates the relationship between Black race and aBW and that the mediation model approaches significance.

## Discussion

The primary aim of these analyses was to model the role of *IGF1* methylation as a mediator of the relationship between race and birth weight. It is well established that Black infants have lower mean birth weights than other racial groups in the United States, even after controlling for gestational age. Our data are consistent with these prior observations. It has been thought that epigenetic changes may mediate the effects of adverse birth outcomes on health outcomes of the offspring. Building on these observations, we found that Black infants had higher *IGF1* methylation than their non-Black counterparts and that *IGF1* methylation was significantly associated with birth weight (Table 3). A formal mediation analysis (Figure 2) suggests that methylation of the *IGF1* gene partially mediates the association between Black race and aBW. Moreover, the magnitude of the indirect effect is moderate in size.

The underlying reason for racial differences in DNA methylation levels, specifically at the *IGF1* locus, at birth is not known. Such methylation differences may reflect variations in the intrauterine environment, including variation by race in maternal or fetal metabolism, genomic signals for methylation, or environmental exposures. Methylation differences by race may also be related to race-specific variation in the distribution of cell types in the mononuclear fraction of cord blood [19]. Differentiating these factors is beyond the scope of our current study. Irrespective of the mechanism, we hypothesize that DNA methylation differences between races may be related to variation in gene expression by race that influence birth weight and, ultimately, risk of disease across the life course.

Consistent with many prior studies suggesting a relationship between variation in *IGF1* expression and birth weight [15,17], our analyses suggest that the higher level of methylation of the *IGF1* was significantly associated with a decrease in aBW (Table 3). While we did not measure *IGF1* expression *per se*, the assessed methylation site was near the transcription start site and might be theoretically expected to result in altered expression. It is important to note that *IGF1* is regulated by two promoters and multiple transcription start sites [20]. The complex structure of *IGF1* makes specific inferences regarding gene expression difficult. Thus additional studies are needed to evaluate how differences in methylation at this and other nearby CpG sites may differentially influence *IGF1* expression generally and by race.

Finally, our analyses suggest that the higher level of methylation of the *IGF1* locus in Black infants accounts for at least part of the disparity in infant birth weight. Most prior research in this area focuses on relations between the two variables considered separately, and much has been written about the relationship between either race or methylation and their effects on birth weight. Our work is the first to formally model the mediation of the relation between race and birth weight by locus specific methylation.

In our model, we specifically hypothesize that *IGF1* methylation is in a causal sequence between race and birth weight. The statistical power to detect mediated effects using these models is typically low [21]. However, while our sample size may not be sufficiently large to achieve statistical significance, analysis of the causal steps in the process using regression models and the formal mediation model suggests that *IGF1* methylation partially mediates the relationship between Black race and aBW [22,23]. That is to say, based on established criteria, partial mediation is likely to be present given the following: the total effect of race on aBW is significant; the effect of race on *IGF1* methylation is significant; the effect of *IGF1* methylation on aBW controlled for race is significant; and, most importantly, the direct effect of race on aBW adjusted for *IGF1* methylation is non-significant and smaller than the total effect. The finding of partial mediation implies that other indirect effects are also likely to have a role in mediating racial differences in birth weight.

## Conclusions

Racial disparities in birth outcomes remain one of the most vexing public health problems in the United States. Our analyses suggest that *IGF1* methylation partially mediates observed racial disparities in birth weight. Our results add to emerging evidence that epigenetic profiles appear to differ across racial groups [10,24,25] and that these differences may contribute to phenotypic differences, such as discordant birth weights. However, birth weight is a complex phenotype influenced by many factors including maternal characteristics, environmental exposures, psychosocial stressors, and infant characteristics. Thus, variation in size at birth results from interaction between maternal genetic factors, fetal genetic factors, the maternal (external) environment, and the intrauterine environment. There may also be effects associated with maternal and paternally derived imprinted fetal genes. While our study did not measure many of these distal factors, our results suggest that they are likely to have a complementary role in mediating racial differences in birth weight. However, differences in DNA methylation and their influence on fetal growth may also represent the end result of the interaction among many of these factors. A more comprehensive examination of the role of epigenetics in the context of these other factors and their influence on racial disparities in perinatal outcomes is warranted.

## Methods

### Study sample and data collection

Data and biological samples were collected as part of a larger study at Tampa General Hospital in Tampa, Florida. The University of South Florida Institutional Review Board approved this study. All infants born at Tampa General Hospital were initially eligible for inclusion into the study; however, infants born to women whose prenatal tests indicated that they were HIV or Hepatitis B positive were excluded. Samples used for this analysis were restricted to live born, singleton infants without known birth defects. Demographic and clinical variables were abstracted from the medical record using standardized forms as part of the parent study. These variables included the following: gestational age (based on clinical estimate and the date of last menstrual period), infant birth weight, infant gender, pregnancy complications, vitamin use, presence of birth defects, plurality, parity, gravidity, prenatal care usage (adequate or not as recorded in the medical record), maternal age, and race (classified as Black or non-Black). Umbilical cord blood samples were collected after delivery into standard EDTA collection tubes. The mononuclear fraction was isolated *via* Ficoll-Paque density gradient centrifugation within 24 hours of sample collection. After separation, the mononuclear layer was suspended in fetal bovine serum and 10% DMSO and stored at −80°C until analysis.

### DNA isolation and methylation analysis

DNA isolation and methylation assessment were conducted by the Wayne State University Applied Genomics Technology Center. DNA was isolated from the mononuclear fraction of umbilical cord blood using the Qiagen EZ1 DNA tissue kit (Qiagen, Valencia, CA, USA) according to the method of Lum et al. with the exception that PBS was substituted for TE buffer [26]. Bisulfite modified DNA was prepared using the EZ-96 DNA Methylation Kit™ (Zymo Research Corp., Irvine, CA, USA) according to the manufacturer’s instructions. Quantitative, loci-specific methylation was assessed using the Infinium HumanMethylation27 BeadArray (Illumina Inc., San Diego, CA, USA) *per* the manufacturer’s instructions.

The array interrogates 27,578 loci located in more than 14,000 genes. For each CpG (cytosine-guanine dinucleotide) site, two different probes (one against the methylated site and one against the unmethylated site) were hybridized with the bisulfite-modified DNA. Next, a single-base extension added one of two possible fluorescent probes (one for methylated (C) and one for unmethylated (T) alleles). Methylation status was then represented by a beta value which is calculated from the ratio of fluorescent signals from methylated to the sum of methylated and unmethylated probes and ranges from 0 (unmethylated) to 1 (methylated). Background normalization was done using the GenomeStudio Methylation module according to the guidelines recommended by Illumina. In short, this method subtracts the average signal of the negative control bead types from the probe signals. Normalized beta values were then output for use in subsequent analyses. In this study, our *a priori* hypotheses focused on the methylation status of *IGF1*; therefore, we analyzed one CpG locus associated with this gene. The nucleotide position of the *IGF1* CpG locus was chr12:101,398,416 according to the NCBI build 36.1.

A subset of samples was run in duplicate in order to assess inter-chip variability. In addition, CpGenome Universal Methylated DNA was used as a positive control (Millipore, Temecula, CA, USA) and was bisulfite treated and run with the methylation assay. The positive control was used to ensure bisulfite conversion and accuracy of methylation measurement. The positive control DNA was almost completely methylated as expected. Inter-chip variability was assessed and was found to be highly reproducible. Pearson correlation coefficients were greater than 0.99 for each set of replicates (*P* < 0.0001). Internal validity was assessed by examining gender specific methylation of six X-linked housekeeping genes (*EFNB1*, *ELK1*, *FMR1*, *G6PD*, *GPC3*, *GLA*) [27,28]. Overall, methylation of the six aforementioned housekeeping genes was as expected in that females exhibited hemimethylation and males had very little methylation at the loci in these genes (*P* < 0.0001 for each gene).

Locus-specific methylation of the *IGF1* CpG site was validated on a subset of the original sample (*N* = 61) using pyrosequencing. EpigenDX (Worcester, MA, USA) designed and conducted the pyrosequencing assay according to manufacturer’s instructions. The correlation between the two measures of methylation (pyrosequencing and the Infinium HumanMethylation27 BeadArray) was evaluated using Pearson’s correlation coefficient. The two measures of methylation were significantly correlated (*r* = 0.36, *P* = 0.005).

### Statistical analyses

SAS version 9.2 (SAS Institute, Cary, NC, USA) was used to perform all analyses. All hypothesis tests were two-tailed with a type 1 error rate fixed at 5%. Demographic and health characteristics were compared using Fisher’s exact test, *t*-tests, and the Wilcoxon-Mann–Whitney test as appropriate.

Multiple linear regression was used to examine DNA methylation as a mediator of the association between race and birth weight. Thus, the main outcome of interest was birth weight. Since birth weight varies significantly with gestational age, birth weight was regressed onto gestational age. The residual values from each fit were added to the mean birth weight and used to represent the gestational aBW. The gestational age-adjusted birth weight variable did not initially meet all the assumptions of linear regression; therefore, it was log transformed to achieve normality. The log-transformed aBW was ultimately used as the main outcome variable and is interpreted as the percent change (100*coefficient) in aBW for a one-unit increase in the dependent variable. A secondary outcome, methylation of *IGF1* was also examined in relation to race. In order to simplify interpretation, the Illumina beta values for *IGF1* methylation were converted to z-scores. Potential confounders were included in the adjusted models if the point estimate changed by more than 10% after adjusting for the confounder.

Mediation analysis was conducted using the methods and macro developed by Valeri and VanderWeele [18]. This framework allowed for the decomposition of a total effect into direct and indirect effects, so that we could statistically test mediation of the race-aBW relationship by *IGF1* methylation. This method uses a model based on two regression equations representing relationships given in Figure 2B:

1 $$ M={y}_0+\alpha (x)+\delta (c)+{e}_0 $$

2 $$ Y={y}_1+\chi (x)+\beta (m)+\delta \hbox{'}(c)+{e}_1 $$

where *Y* is the dependent variable (log-transformed gestational age-adjusted birth weight), *x* is the independent variable (race), *M* is the mediator (*IGF1* methylation status), *y*_0_ and *y*_1_ are intercepts, *e*_0_ and *e*_1_ are the corresponding residuals, and *c* are covariates. In this model, *χ* is defined as the controlled direct effect relating the independent variable (race) to the dependent variable (log(aBW)) adjusted for the mediator (*IGF1* methylation status) and relevant covariates. The natural indirect effect is the product *α × β* that is used to test the statistical significance of the mediation model.

An important property of the natural indirect effect and the controlled direct effect is that they can be related to the total effect (Figure 2A), defined as

3 $$ Y={y}_1+\chi \hbox{'}(x)+\delta \hbox{'}(c)+{e}_2 $$

which relates the independent variable (race) and the dependent variable (log(aBW)), such that

*α* × *β* is equivalent to *χ* ’ − *χ* (the difference between the total effect and the controlled direct effect).
